# PrOnto database : GO term functional dissimilarity inferred from biological data

**DOI:** 10.3389/fgene.2015.00200

**Published:** 2015-06-03

**Authors:** Charles E. Chapple, Carl Herrmann, Christine Brun

**Affiliations:** ^1^Inserm, UMR_S1090 TAGCMarseille, France; ^2^Aix-Marseille Université, UMR_S1090 TAGCMarseille, France; ^3^Centre National de la Recherche ScientifiqueMarseille, France

**Keywords:** moonlighting protein, gene ontology, functional similarity, protein-protein interactions, database

## Abstract

Moonlighting proteins are defined by their involvement in multiple, unrelated functions. The computational prediction of such proteins requires a formal method of assessing the similarity of cellular processes, for example, by identifying dissimilar Gene Ontology terms. While many measures of Gene Ontology term similarity exist, most depend on abstract mathematical analyses of the structure of the GO tree and do not necessarily represent the underlying biology. Here, we propose two metrics of GO term *functional dissimilarity* derived from biological information, one based on the protein annotations and the other on the interactions between proteins. They have been collected in the PrOnto database, a novel tool which can be of particular use for the identification of moonlighting proteins. The database can be queried via an web-based interface which is freely available at http://tagc.univ-mrs.fr/pronto.

## 1. Introduction

Moonlighting proteins are a subset of multifunctional proteins involved in several, unrelated biological functions. Because of the growing importance of this functional singularity (Copley, 2012) for the understanding of cellular regulations and human diseases (Jeffery, 2011), computational methods for the large scale prediction of moonlighting proteins have long been awaited (Khan and Kihara, 2014). Yet, so far, most of the known moonlighting proteins were serendipitous discoveries (Mani et al., 2015). One of the major hurdles that need to be overcome in order to tackle such a task is defining the notion of “unrelated functions.” What are biologically “unrelated functions” in the context of moonlighting? How can they be defined according to the current gene/protein functional annotations in way that computers can understand?

The Gene Ontology (GO) (Ashburner et al., 2000) is a controlled vocabulary of terms to describe gene product functions. Over the last decade, it has become the *de facto* standard ontology used to formalize gene annotation data. It is organized as three independent directed acyclic graphs (DAGs), one for each of the sub-ontologies Biological Process (BP), Molecular Function (MF), and Cellular Component (CC).

The structure of the GO DAG means that many GO terms are related, either because they describe related functions or because one term is the child of another. Therefore, proteins annotated to similar GO terms are assumed to perform similar functions and can be categorized as such. This has led to various methods of evaluating the semantic similarity of GO annotations (reviewed in Gan et al., 2013). Most of these depend on the relationships between the terms in the DAG, either by measuring their distance as the number of edges connecting them, or by evaluating their information content. Such methods can therefore identify semantically similar GO terms, cases where the terms are linked in the structure of the DAG. The identification of moonlighting proteins requires defining *dissimilar functions*. However, *semantically dissimilar* GO terms are often clearly connected from a biological perspective, and therefore semantic similarity measures are not the best option for implementation in a moonlighting discovery pipeline. For instance, the terms “response to tumor necrosis factor” (GO:0034612) and “positive regulation of apoptotic process” (GO:0043065) share no parent terms apart from the root of the ontology although they are descriptions of tightly linked biological processes. Indeed, TNF is a well known inducer of apoptosis (see Gaur and Aggarwal, 2003 for a review) and “positive regulation of apoptotic process” describes one of the cellular “responses to tumor necrosis factor.” These *semantically dissimilar* terms can, therefore, be considered *functionally similar* since they are different descriptions of the same or obviously connected biological processes. This similarity is reflected in the fact that the two terms co-occur in the annotations of multiple proteins (e.g., 19 and 25 in the mouse and human proteomes, respectively).

**Figure 1:**
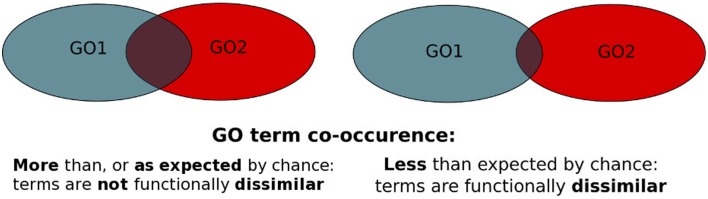
**PrOnto probabilities principle**. The blue set represents the proteins annotated to GO1, the red set represents proteins annotated to GO2. The intersection corresponds to either proteins annotated to both terms (*APs*) or interactions involving proteins annotated to both terms (*IPs*).

We have therefore developed PrOnto, a web-based tool that provides two metrics of GO *functional dissimilarity* based on gene product GO annotations and protein-protein interactions (PPI). We use the frequency of co-occurrence of GO term pairs in (i) protein annotations (Annotation Probabilities, *APs*) and in (ii) the annotations of interacting protein pairs (Interaction Probabilities, *IPs*) to compute probabilities reflecting biases toward infrequent GO terms associations implying *functional dissimilarity* (Figure 1). In this paper, we present the metrics, the webtool we provide to the community as well as different usage examples among which our recent characterization of potential moonlighting proteins from the human PPI network (Chapple et al., 2015).

**Figure 2:**
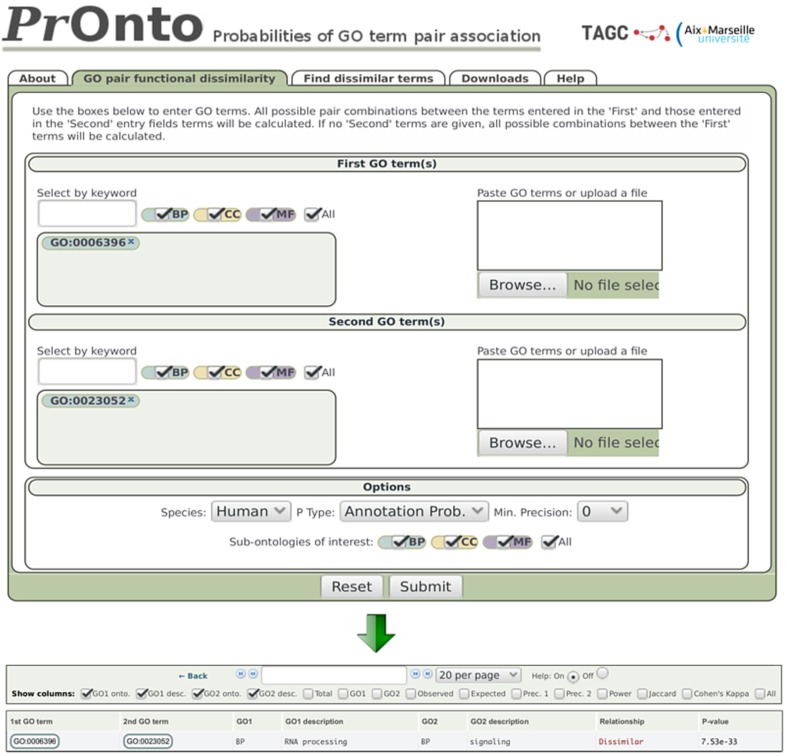
**PrOnto web interface**. In the example shown, two terms have been submitted. The lower panel shows the results for *APs*.

The current version of the PrOnto database contains probabilities for human, mouse, fly, worm and yeast (see Supplementary Table 1 for database statistics). The database will be regularly updated to keep up with annotation and interaction data. PrOnto is free and accessible through a simple web-based interface (see Figure 2 available at http://tagc.univ-mrs.fr/pronto).

## 2. Materials and methods

### 2.1. Annotation and interaction probabilities

We express *functional dissimilarity* as a probability of GO term pair co-occurrence modeled by a hypergeometric distribution. Let *X* be the variable “number of proteins annotated to both terms” which follows a hypergeometric law. The probability of observing this or smaller values of *X* by chance is given by

(1) $$P(X\leq k_{0})=\sum_{k\;=\;0}^{k_{0}}P(X=k)=\sum_{k=0}^{k_{0}}\frac{\left(\begin{array}{c} K\\ k \end{array}\right)\left(\begin{array}{c} N-K\\ n-k \end{array}\right)}{\left(\begin{array}{c} N\\ n \end{array}\right)}$$

with

(2) $$\;P(X=0)=\frac{\left(\begin{array}{c} N-K\\ n \end{array}\right)}{\left(\begin{array}{c} N\\ n \end{array}\right)}$$

where, for *AnnotationProbabilities* (*APs*), *N* is the number of proteins annotated directly to at least two different GO terms, *K* is the number of proteins annotated to *GO*_1_, *n* is the number of proteins annotated to *GO*_2_ and *k* is the number of proteins annotated to both terms.

For *InteractionProbabilities* (*IPs*), *N* is the number of interactions in the PPI network between proteins annotated directly to at least two different GO terms. *K* is the number of interactions involving proteins annotated to *GO*_1_, *n* the number of interactions involving proteins annotated to *GO*_2_ and *k* the number of interactions between a protein annotated to *GO*_1_ and one annotated to *GO*_2_.

In both cases, *N* is the size of the event pool. *P*(*X*) is the probability of observing as large a co-occurence as *X* in a set of size *N*. Therefore, when computing *N*, only proteins with at least 2 direct annotations (i.e., explicit annotations, not including the implicit parent terms) are considered since co-occurence is only relevant for proteins annotated with at least two different GO terms. When calculating cross-ontology probabilities, only proteins with at least one explicit annotation in each ontology of interest are considered. When calculating *K*, *n*, and *k*, all annotations per protein are counted, both direct and inherited. All GO annotations have been included, irrespective of their evidence codes. Indeed, electronic annotations have greatly improved in recent years and their reliability now rivals that of manual annotations (Skunca et al., 2012). Pairs whose low-tail *p*-value is below a user-defined threshold (default: 0.05) are listed as “Dissimilar” and all others as “Not dissimilar.”

In addition, the Jaccard index and Cohen's kappa have been computed for all pairs.

### 2.2. Building high quality PPI networks

To calculate the *IPs*, a high quality interactome was compiled for each target species. Interaction data were retrieved using the PSIQUICK (Aranda et al., 2011) interfaces of the APID (Prieto and Rivas, 2006), BioGrid (Chatr-Aryamontri et al., 2013), IntAct (Kerrien et al., 2012), DIP (Salwinski et al., 2004), MINT (Ceol et al., 2010), MatrixDB (Chautard et al., 2009), Reactome (Croft et al., 2011), InnateDB (Lynn et al., 2008), MolCon, Spike (Elkon et al., 2008), and TopFind (Lange and Overall, 2011) databases. Interaction data were filtered by identification method and only binary interactions between proteins were kept. A full list of the PSI-MI IDs used to build out networks is provided as Supplementary Table 2.

Protein names were mapped to UniProt IDs, and sequences, downloaded from UniProt, clustered using CD-HIT (Fu et al., 2012). TrEMBL/SwissProt protein pairs sharing ≥95% similarity were considered to be the same protein: interactions of the TrEMBL protein were then inherited by the Swiss-Prot protein. Self interactions were discarded.

The final result was high quality interactomes consisting entirely of experimentally verified, direct, binary interaction pairs for each species studied. These networks will be regularly updated with each update of PrOnto. Current interactomes are available on the downloads page of the PrOnto database (http://tagc.univ-mrs.fr/pronto/index.php?id=downloads).

### 2.3. Tools and resources used

The probabilities were calculated using the phyper function of the R statistical environment (R Core Team, 2012) and protein annotations were taken from the EBI's QuickGO service (Barrell et al., 2009) (ftp://ftp.ebi.ac.uk/pub/databases/GO/goa/). The power of the test was calculated using the power.fischer.test function of the statmod R package and 1000 simulations per pair. Jaccard indeces and Cohen's kappa were calculated using a simple awk script. The PrOnto webpage is written using a combination of HTML, PHP, and Javascript, the data are stored in a MySQL database which is queried using a Perl script.

## 3. Results and discussion

### 3.1. PrOnto content: the different PrOnto categories

PrOnto probabilities have been computed from the proteomes and interactomes of several species (human, mouse, fly, worm, yeast) to assess the relationships between GO terms of the same sub-ontology as well as between sub-ontologies.

***Dissimilar*** - When the low-tail *p* < 0.05, the GO terms are very rarely associated among protein annotations and are therefore qualified as “dissimilar” by PrOnto. For example, the probability of finding a lower co-occurrence than observed for the GO terms “RNA processing” (GO:0006396) and “signaling” (GO:0023052) is very low in human (therefore highly significant), as indicated by their *AP*, *p* = 7.5e-33 (Figure 2). The co-occurrence is even less probable between interacting proteins since for *IPs*, *p* = 1.4e-230. These GO terms are then functionally “dissimilar” and very rarely linked through protein-protein interactions. Interestingly, these processes have been shown to be instead linked through protein-RNA interactions (Hogan et al., 2008). To demonstrate the validity of our approach when identifying dissimilar pairs, the power of the hypergeometric test was calculated for all pairs for which the null hypothesis was not rejected (dissimilar pairs). The results are shown in Supplementary Figure 1. Notably, the power was very high for the overwhelming majority of pairs (mean = 0.90 and median = 0.99).

***Not Dissimilar*** - When the *p* ≥ 0.05, PrOnto returns *Not Dissimilar*. For instance, in the human proteome, the probability of finding a greater co-occurrence than observed for the terms “response to tumor necrosis factor” (GO:0034612) and “positive regulation of apoptosis” (GO:0043065) is low (*AP p* = 1, therefore highly significant), clearly indicating that the terms are functionally “similar.” This is explained by the following: as 116 human proteins are annotated to “response to tumor necrosis factor” and 459 to “positive regulation of apoptosis,” 3 are expected to be annotated to both given the human proteome size (17866 annotated proteins), but 25 co-annotated proteins are observed. The same is observed for *IP* as proteins that respond to TNF signaling will often interact with those that promote apoptosis.

***NA*** - Additionally, a *NA* category exists when no score could be computed. PrOnto produces *NA* when at least one of the GO terms of the pair under consideration does not annotate any protein in the target species. For instance, PrOnto returns “NA” for the GO terms GO:0030326 (“embryonic limb morphogenesis”) and GO:0048736 (“appendage development”) in yeast. This makes perfect sense biologically speaking since, for obvious reasons, no yeast proteins will be annotated to either of those terms.

Globally, as shown in Table 1, where the percentages of dissimilar and not dissimilar GO term pairs are reported for each species, most GO term pairs are not dissimilar according to PrOnto (*AP*, 84.1–87.1% and *IP*, 54.2–72.3%). Since the cell is a complex system whose constituent parts are very often interlinked (Schwikowski et al., 2000), many GO terms co-occur more often than expected by chance among gene/protein annotations or between interacting proteins. This link between processes in the cell is thus captured by PrOnto which is based on functional data.

**Table 1:** **Percentage of GO term pairs from all ontologies (including cross-ontology pairs) that are Dissimilar or Not Dissimilar for all species**.

	**AP**		**IP**	
	**Dissimilar**	**Not dissimilar**	**Dissimilar**	**Not dissimilar**
Human	0.3	99.6	4.0	96
Mouse	0.2	99.8	0.5	99.6
Fly	0.5	99.5	2.2	97.8
Worm	0.6	99.4	2.2	97.8
Yeast	0.5	99.5	4.9	95.2

*“Dissimilar” corresponds to low-tail *p* < 0.05 and “Not Dissimilar” to all other cases*.

As expected, since PrOnto is based on existing protein annotations, dissimilar terms according to PrOnto are rare (*AP*, 0.2–0.6%). That they are more numerous in the *IPs* (0.5–4.9%) shows functions that are rarely carried out by interacting proteins, therefore suggesting that they are performed by different functional modules, known as groups of interacting proteins involved in the same biological process (Spirin and Mirny, 2003). Interestingly, a larger proportion of dissimilar pairs has been identified in the organisms for which interaction data are more complete (human and yeast), highlighting the necessity of deciphering protein-protein networks in other organisms to gain a deeper understanding of the links between functions.

Table 2 shows the percentage of dissimilar GO term pairs per ontology for human. Interestingly, the fact that dissimilar terms reach 7% for *IP* CC pairs reflect the shuttling of proteins throughout different cell compartments.

**Table 2:** **Percentage GO term pairs from each ontology, excluding cross-ontology, for human (MF: Molecular Function, CC: Cellular Component, BP: Biological Process) that are Dissimilar or Not Dissimilar**.

	**AP**		**IP**	
	**Dissimilar**	**Not dissimilar**	**Dissimilar**	**Not dissimilar**
MF	0.4	99.6	2.0	98
CC	1.3	98.6	7.0	93.1
BP	0.3	99.8	4.2	95.7

*“Dissimilar” corresponds to low-tail p < 0.05 and “Not Dissimilar” to all other cases*.

### 3.2. PrOnto usage to predict moonlighting protein candidates

Unlike most tools that provide a measure of GO term functional association, PrOnto was conceived in order to identify dissimilar terms. Indeed, whereas most comparable approaches are geared toward the identification of similar terms, PrOnto has the advantage of being able to identify terms that very rarely co-occur. We therefore used PrOnto probabilities in a protein-protein network analysis dedicated to the discovery of moonlighting candidates and implemented them in our MoonGO pipeline (Chapple et al., 2015). The pipeline first extracts overlapping clusters from a PPI network using the OCG algorithm (Becker et al., 2012). These clusters are formed by highly interconnected proteins which tend to be involved in the same cellular processes. The cellular process(es) in which the clusters are involved, are identified based on the BP GO annotations of their constituent proteins, following a majority rule. Potential moonlighting proteins are then identified at the intersection of clusters involved in unrelated biological processes according to PrOnto GO term association probabilities. Using both APs and IPs ensures that the multiple functions in which the candidate protein is found to be involved, are very rarely performed (i) by a single protein and (ii) by interacting proteins. APs and IPs are therefore used here as two proxies, indicators of unrelated cellular functions.

Using this approach, we have identified 430 moonlighting candidates that form a distinct sub-group of proteins displaying specific features, distinguishing them from non-candidates proteins and constituting a signature of extreme multifunctionality. Among the striking features, candidates are more connected in the network, enriched in short linear motifs and in disease-related proteins compared to non-candidates, and are less intrinsically disordered than network hubs (Chapple et al., 2015; Zanzoni et al., 2015). These results therefore underline that PrOnto is particularly well suited to identify moonlighting candidates from biological data since it is especially stringent when determining term dissimilarity (see Tables 1, 2).

As we provide PrOnto probabilities for multiple species, we predict that it will be soon used for the identification of moonlighting candidates in these other species. Finally, it should be noted that a recent analysis of GO terms has been proposed to identify moonlighting candidates in *E. coli* (Khan et al., 2014). Unlike our approach which uses PrOnto probabilities to assess the dissimilarity of functions of functional modules, this work is comparing the extent to which each function of the moonlighting candidates is described by the GO terms annotating the proteins, using a semantic similarity measure. The approach is therefore also using GO term annotations but in a completely different context.

### 3.3. Other PrOnto uses

Overall, *APs* can be used to identify cellular processes that are rarely carried out by the same proteins (i.e., “dissimilar”), whereas *IPs* can offer insights into the links between different cellular processes mediated by protein interactions. In addition their use for the investigation of the links between the functional modules formed by interacting proteins in PPI networks as described above, *APs* and *IPs* could also be used to score protein interaction predictions. In some cases, in the absence of experimental data, protein-protein interactions are predicted using bioinformatics approaches. One may then want to assign a confidence score based on real biological data to these predictions. Because PrOnto probabilities are derived from biological knowledge and experimental data, they can be used for this purpose and a lower score can be assigned to predicted interactions between proteins annotated to dissimilar terms.

They can also be used when constructing ontologies from -omics data as recently proposed by Dutkowski et al. (2013) and Kramer et al. (2014), the latter of which uses semantic similarity for assessment purposes. PrOnto can also help guide protein annotations as in the annotation tool GOAT (Bada et al., 2004) which uses term functional association scores to annotate proteins of unknown function. Once one term has been assigned to a protein, dissimilar terms are less likely to be added.

### 3.4. PrOnto compared to similar tools

Using term co-occurence as a proxy for functional similarity has already been done by other methods and tools. For example, the EBI's QuickGO service provides the top 100 most co-occuring terms for the query GO term. However, a way of getting more than those 100 co-occuring terms is not provided, nor is any way of getting terms that do not co-occur (the “dissimilar” terms of PrOnto). As already mentioned, the later can be very useful in studies of protein multifunctionality.

The FAM (Function Association Matrix) which is part of the PFP protein function predictor (Hawkins et al., 2009) uses a very similar approach. However, the FAM scores (available at http://dragon.bio.purdue.edu/FAM/), unlike PrOnto, are asymmetric, meaning that *P*(*GO*1|*GO*2) ≠ *P*(*GO*2|*GO*1) which is something that should be taken into account when choosing which tool to use. Depending on the analysis, either symmetric or asymmetric probabilities might be preferred.

In addition, both methods (QuickGO and FAM) provide co-occurrences calculated from the entirety of the UniProt database whereas PrOnto considers a specific species' proteome or interactome. A species-specific measure can indeed be considered an asset depending on the analysis being undertaken since cellular and physiological functions often differ between species, essentially due to tissue-specificity. Term co-occurences are therefore expected to be different across species.

Moreover, neither method provides the user with the ability to input a list of terms and obtain a list of probabilities nor the possibility of querying for specific pairs. The web-based interface of PrOnto is designed with that in mind and is a simple and practical way of quantifying GO term functional dissimilarity.

Finally, PrOnto is, to our knowledge, the only tool that offers a measure of GO term functional dissimilarity based on a species' interactome. A similar approach was undertaken by Dotan-Cohen et al. (2009) to identify “Process Linkage Networks” rather than to assess GO term functional dissimilarity. While they used interactome data of a single species to build these networks, they did not provide a tool to the community.

## 4. Conclusion

We have presented PrOnto, a novel tool for quantifying GO term *functional dissimilariy* based on two species-dependent metrics of GO term association derived from either the annotations or the interactome data of the species in question. This tool was developed for the specific goal of identifying moonlighting proteins from interactome data. As such, the emphasis has been on the identification of dissimilar functions.

The current version of PrOnto is using a relatively simple statistical approach which, nevertheless provides robust results (see Chapple et al., 2015 and Supplementary Figure 1). In the future, in addition to the hypergeometric probability, power and Jaccard indeces, we plan to implement other statistical measures and combine them into a single metric of GO term similarity. In addition, we plan to expand the tool to also address “similar,” as opposed to merely “not dissimilar,” GO terms.

### Conflict of interest statement

The authors declare that the research was conducted in the absence of any commercial or financial relationships that could be construed as a potential conflict of interest.

## Supplementary material

Supplementary Figure 1 **Density plot of the power of the hypergeometric test for all GO pairs tested**. The power is high for the vast majority of pairs tested (mean = 0.90 and median = 0.99).

Supplementary Table 1 **The number of GO pairs for each of the possible sub-ontology combinations**. CC, Cellular Compartment; BP, Biological Process; MF, Molecular Function.

Supplementary Table 2 **List of experimental methods identifying binary protein-protein interactions**.
