# Supplementary figures and images for: PrOnto database : GO term functional dissimilarity inferred from biological data
Source: Front Genet. 2015 Jun 3;6:200. doi: 10.3389/fgene.2015.00200 (PMC4452890; doi:10.3389/fgene.2015.00200)

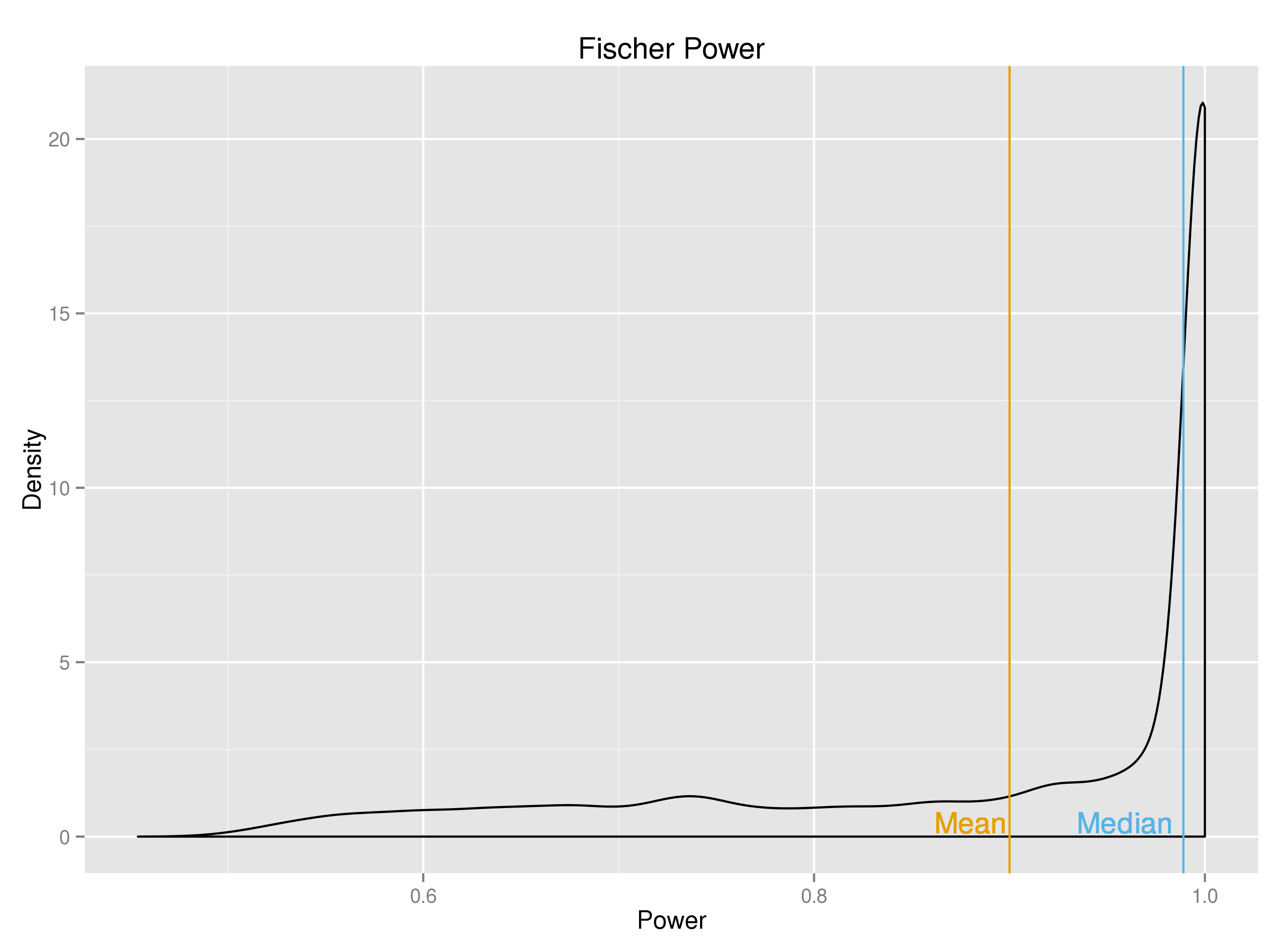

Supplement: Supplementary Figure 1 — Density plot of the power of the hypergeometric test for all GO pairs tested. The power is high for the vast majority of pairs tested (mean = 0.90 and median = 0.99). [file Image1.PNG]
