# Supplementary material for: PrOnto database : GO term functional dissimilarity inferred from biological data
Source: Front Genet. 2015 Jun 3;6:200. doi: 10.3389/fgene.2015.00200 (PMC4452890; doi:10.3389/fgene.2015.00200)
Supplement: Supplementary Table 1 — The number of GO pairs for each of the possible sub-ontology combinations. CC, Cellular Compartment; BP, Biological Process; MF, Molecular Function. [file Table1.PDF]

Table S1. The number of GO pairs for each of the possible sub-ontology combinations

Annotation Probabilities

	CC/CC	CC/MF	CC/BP	MF/MF	MF/BP	BP/BP
human	932,295	5,376,576	16,508,110	7,744,080	47,566,560	73,017,570
fly	406,351	2,133,230	5,156,734	2,795,430	13,520,705	16,339,186
mouse	873,181	4,981,296	15,463,434	7,097,028	44,074,296	68,404,056
yeast	353,220	1,858,610	3,633,961	2,440,945	9,549,410	9,333,360
worm	260,281	1,337,866	2,713,276	1,715,878	6,963,574	7,059,403

Interaction Probabilities

	CC/CC	CC/MF	CC/BP	MF/MF	MF/BP	BP/BP
human	869,221	4,499,652	15,122,732	5,829,405	39,172,236	65,832,075
fly	365,085	1,713,978	4,397,246	2,015,028	10,334,043	13,258,675
mouse	403,651	1,441,290	6,835,576	1,288,815	12,217,260	28,975,078
yeast	350,703	1,778,625	3,574,827	2,258,875	9,075,875	9,122,856
worm	171,405	687,960	1,678,365	692,076	3,373,944	4,117,015
